# Can social dancing prevent falls in older adults? a protocol of the Dance, Aging, Cognition, Economics (DAnCE) fall prevention randomised controlled trial

**DOI:** 10.1186/1471-2458-13-477

**Published:** 2013-05-15

**Authors:** Dafna Merom, Robert Cumming, Erin Mathieu, Kaarin J Anstey, Chris Rissel, Judy M Simpson, Rachael L Morton, Ester Cerin, Catherine Sherrington, Stephen R Lord

**Affiliations:** 1 School of Science and Health, University of Western Sydney, Sydney, Australia; 2 School of Public Health, University of Sydney, Sydney, Australia; 3 Centre for Research on Aging, Health and Wellbeing, The Australian National University, Canberra, Australia; 4 Centre for Physical Activity and Nutrition Research, Deakin University, Melbourne, Australia; 5 The George Institute for Global Health, University of Sydney, Sydney, Australia; 6 Neuroscience Research Australia (NeuRA), University of New South Wales, New South Wales, Sydney, Australia

## Abstract

**Background:**

Falls are one of the most common health problems among older people and pose a major economic burden on health care systems. Exercise is an accepted stand-alone fall prevention strategy particularly if it is balance training or regular participation in Tai chi. Dance shares the ‘holistic’ approach of practices such as Tai chi. It is a complex sensorimotor rhythmic activity integrating multiple physical, cognitive and social elements. Small-scale randomised controlled trials have indicated that diverse dance styles can improve measures of balance and mobility in older people, but none of these studies has examined the effect of dance on falls or cognition. This study aims to determine whether participation in social dancing: i) reduces the number of falls; and ii) improves cognitive functions associated with fall risk in older people.

**Methods/design:**

A single-blind, cluster randomised controlled trial of 12 months duration will be conducted. Approximately 450 participants will be recruited from 24 self-care retirement villages that house at least 60 residents each in Sydney, Australia. Village residents without cognitive impairment and obtain medical clearance will be eligible. After comprehensive baseline measurements including physiological and cognitive tests and self-completed questionnaires, villages will be randomised to intervention sites (ballroom or folk dance) or to a wait-listed control using a computer randomisation method that minimises imbalances between villages based on two baseline fall risk measures. Main outcome measures are falls, prospectively measured, and the Trail Making cognitive function test. Cost-effectiveness and cost-utility analyses will be performed.

**Discussion:**

This study offers a novel approach to balance training for older people. As a community-based approach to fall prevention, dance offers older people an opportunity for greater social engagement, thereby making a major contribution to healthy ageing. Providing diversity in exercise programs targeting seniors recognises the heterogeneity of multicultural populations and may further increase the number of taking part in exercise.

**Trial registration:**

Australian New Zealand Clinical Trials Registry ACTRN12612000889853

The trial is now in progress with 12 villages already have been randomised.

## Background

Public health systems across the world are facing the challenge of greater longevity and consequently increasing numbers of age-related adverse health conditions. Falls are among the most common age-related health problems for older adults in developed countries [1], including Australia [2]. For example, in New South Wales, the most populous Australian state, one in four adults aged 65 years and older fall annually, and 10% of these falls result in hospitalisation [3]. The consequences of a fall even without physical injury are grave: falls can lead to fear of falling, poor quality of life, loss of independence, and nursing home admission [4,5]. The direct health care cost of fall-related injuries was estimated at AUD$558.5 million, or nearly 5% of the total health care budget of NSW, in 2006/7 and it is expected to triple by 2050 based on demographic shifts alone [6], unless effective preventive strategies are put in place.

Exercise is an accepted stand-alone strategy which addresses the physiological deficits that are part of the multi-factorial aetiology of falls; systematic reviews report that exercise programs reduce fall rates by an average of 16% [7,8]. As a result, exercise interventions now form part of clinical guidelines for fall prevention in the United States (US), United Kingdom (UK) [9] and Australia [10]. Most programs target fitness dimensions (e.g., muscle strength, aerobic capacity, balance, agility) are delivered (a) with a focus on one dimension only, or (b) in combination, but in a segmented ‘stop-start’ manner. These approaches may not be the best ways to optimise benefits. For example, Tai chi interventions have been shown to reduce the risk of falls by 37% compared to 22% in multi-component group-based exercise [8]. Tai chi integrates multiple physical and cognitive elements that are claimed to be synergetic and involve the whole “body-mind system” rather than a composite of separate components [11].

Dance also shares the ‘holistic’ practice approach. It is a complex sensorimotor rhythmic activity integrating multiple physical, cognitive and social elements, all of which have the potential to ameliorate a wide range of physiological and cognitive fall risk factors. Furthermore, unlike exercise programs that require training for specialised instructors, dancing is already available in the community and therefore inherently sustainable.

The potential of dance as a promising alternative to existing fall prevention exercise was first mooted in the public health literature in 2003, albeit with limited empirical support, mostly inferred from studies of exceptional balance abilities of professional young dancers [12]. Since then, an increasing number of dance-based studies involving older adults have been published [13], supporting the benefits of dance in improving impaired gait and balance, two of the strongest risk factors for falls in older people [14]. For example, cross-sectional studies have shown that seniors who dance have superior balance and gait characteristics compared to aged-matched controls [15,16]. Small-scale (≤50 participants) randomised controlled trials (RCTs) have also shown that a variety of dance styles result in improved balance and gait speed of older people [17-21]. Yet, none of the intervention studies, whether RCTs or quasi-experimental [22-25], examined whether these programs also reduced the incidence of falls or addressed cognitive risk factors for falls [5,26-28].

This study aims to determine whether a typical community social dance program i) reduces the number of falls; and ii) improves cognitive factors associated with fall risk in older people. We hypothesise that dance will lead to at least a 37% reduction in the incidence of falls, based on the pooled effect size of Tai chi trials, [8] and that its effect on cognitive function will be at least as strong as that resulting from other aerobic training (e.g., walking on treadmill) interventions [29].

## Methods

### Design

**Figure 1:**
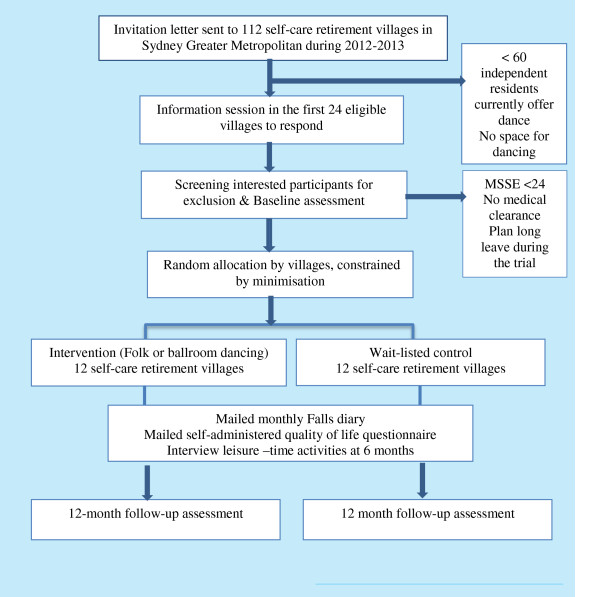
Study flow chart.

A single-blind, cluster randomised controlled trial, will be conducted in self-care retirement villages (clusters) as described in the study flow chart (Figure 1). Ethics approval has been granted by the Human Research Ethics Committee (ref: 9468) University of Western Sydney and the trial has been registered with the Australian and New Zealand Clinical Trials Register (ACTRN12612000889853).

### Recruitment

Independent care residents of participating villages will be invited to attend an information session. Residents will be made aware of the information session via their village’s internal mail system and other channels (e.g. notice boards, village newsletters). At the information session, interested participants will provide researchers with their contact details and receive information sheets and consent forms. Those not interested in participating will be asked to complete a short questionnaire giving their age, sex and reasons why they declined participation. Residents who are interested in participating, but unable to attend the information session, will be provided with researchers’ contact details by village staff.

### Eligibility criteria

Eligible retirement villages will be those in the metropolitan area of Sydney, Australia, that: have at least 60 independent (self-care) residents; have a common facility area suitable for dance classes; and are not offering dance classes. A minimum of 12 participants from each retirement village will be recruited. Eligible participants in the trial must: be a resident of the village; agree to undergo physical and cognitive testing; and obtain medical clearance to participate in the study. Participants will be excluded if they: score less than 24 on the Mini Mental State Examination (MMSE) indicating cognitive impairment [30]; or plan to leave the village for three months or more during the trial.

### Screening for eligibility and baseline data collection

Researchers will contact interested participants in order to conduct a short telephone interview and to schedule a time for baseline measurement at the retirement village. During the baseline measurement session, researchers will first administer the MMSE to determine eligibility. Baseline measures will then be collected from eligible participants by up to three trained researchers. All participants will self-complete a questionnaire, and will undergo a series of physiological and psychological examinations as detailed below:

• The baseline questionnaire includes questions and scales addressing: demographics, physical activity [31], other leisure pursuits (e.g., reading, attending social clubs, games, cultural activity) [32] medical history, fall history, falls self-efficacy [33], quality of life assessments [34], depressive symptoms (15-item Geriatric Depression Scale (GDS)) [35], social networks [36], history of dancing and dance self-efficacy.

• Physiological measurements include the Physiological Performance Assessment (PPA) [37], the Short Physical Performance Battery (SPPB) [38], and Choice Stepping Reaction Time (CSRT) [39] with and without a cognitive load [40].

• Psychological measurements include the Trail Making Tests (TMT) part A and B [41] and the Rey Auditory Verbal Learning test (RAVLT) [42]. These tests are described in detail in the main outcomes section.

### Randomisation and allocation concealment

After completion of the baseline measures and obtaining all medical clearances, the study coordinator will provide the study statistician with village means of two baseline tests that predict falls and recurrent falls, the PPA [37] and TMT part B [5]. Retirement villages will be randomised to intervention or control group by the study statistician using a computer generated randomisation method, constrained using minimisation. That is, each village will be allocated with a probability of 0.8 to the group that minimises the imbalance of the means of these two baseline variables between intervention and control groups, using two strata for each variable. The statistician will advise the study coordinator of the village’s allocation and the study coordinator will arrange for delivery of the intervention if appropriate. Allocation will thus be concealed from the research team recruiting villages and participants, as randomisation will occur after baseline measures have been completed. Villages and participants will be considered to have entered the trial at the point of randomisation.

#### Intervention

Participants in the intervention villages will be offered 80 hours of dance classes during a 12-month period. Dance classes will run for one hour, twice a week for approximately 40 weeks (allowing for short breaks during the 12-month period). Intervention villages will be offered two major styles: 1) Folk dancing which will include a collection of dances from the US, UK, France, Italy, Israel and Greece; and 2) ballroom dancing which will include a collection of dances such as Rock and Roll, Foxtrot, Waltz, and Latin dances including Salsa and Rumba. Dance programs will not be manipulated to mimic exercise training as in aerobic dance classes, nor will they incorporate any specific strength or balance training as reported in a pilot RCT [17]. The aim is to test the efficacy of dance programs as offered in the community. However the intervention will be standardised across all sites via periodical workshops, a guidebook and a DVD developed by the dance coordinators. The dance classes will be taught at varying levels of complexity and the progression during the year will be gradual. Progression to new dances will take into account the balance challenge, cardiovascular demand, degree of flexibility, coordination and cognitive demand. Dance intensity will be monitored using accelerometers three times during the 12-month period (after 20, 40 and 60 hours of dance classes).

Retirement villages and participants in the control group will be advised to continue with their regular activities, and asked not to join a dance class during this time. Controls will be placed on a wait list for the dance classes.

All participants in the study will be sent monthly newsletters containing study updates, a “meet the team” section and a health education section (e.g., vitamin D, sleeping habits, healthy eating, smoking cessation, appropriate footwear, reducing alcohol intake, falls statistics and the origin of ballroom/folk dance). Further information contained in the monthly newsletters can be obtained by contacting the corresponding author.

### Primary and secondary outcomes and measures

The primary outcomes are:

1) Number of falls

2) Time to complete TMT parts A and B

The secondary outcomes are:

1) PPA score

2) CSRT with and without cognitive load

3) Lower limb functional status

4) Quality of Life

5) Cost per fall prevented

6) Cost per quality adjusted life year (QALY) gained

The details for each of these measures are given below.

*Number of falls during the 12 month trial*. A fall is defined as ‘unintentionally coming to rest on the ground, floor, or other lower level’ [43]. Participants are asked to record falls daily for the duration of the study using a monthly calendar (diary). Participants will be asked to record ‘F’ (Fall) or ‘N’ (no fall) each day on the calendar and return this by mail at the end of each month. Participants who report a fall will be interviewed by telephone to obtain specific details about where the fall occurred, resulting injuries and any treatment sought. Participants who fail to return their calendars within two weeks will be contacted by phone. They will inform the researchers over the phone of the entries in their calendar for the past month, and will also be asked also return their calendar by mail.

*Time to complete TMT Parts A and B*[41]. Part A measures processing speed and involves participants connecting consecutive numbers (e.g., 1-2-3). Part B is a measure of executive function of ‘task shifting’ and involves participants connecting alternating letters and numbers (e.g.,1-A-2-B). The difference in time between the two parts (B minus A) will be calculated to isolate the executive component of this test.

*The PPA* comprises five tests that provide a physiological fall risk score: vision (edge contrast sensitivity), peripheral sensation proprioception, lower extremity strength (knee extension), simple reaction time using a figure press as the response, and balance measured by body sway when standing on a medium-density foam rubber mat.

*CSRT with and without cognitive load*[39,40]. Two tests require the participant to perform quick, correctly targeted steps measured in milliseconds using a dance mat. In its simple form (test 1), participants see a graphical presentation of the four arrows on the mat (front left, front right, side left, side right) on a computer screen. The step direction is indicated by one arrow changing its colour. Participants are asked to step as quickly as possible onto the corresponding arrow of the mat and return to the centre (stance panels). The test consists of 4 practice trials (one trial for each step direction: side left, front left, front right, side right) and 32 test trials with stimuli occurring randomly between 1 and 2 seconds after the participant returns to the centre.

The second test (the Stroop stepping test) measures response inhibition and selective attention. For this test eight panels on the dance mat are used: 2 central stance panels, 2 front panels, 1 left panel, 1 right panel and 2 back panels. In the centre of the computer screen an arrow is presented pointing in one of four directions (up, down, left, right) that match the four possible step directions (forward, backward, left, right). Inside the arrow is a written word indicating a direction different to that indicated by the arrow. Participants have to step according to the word (ignoring the direction of the arrow) and then return to the centre panels. After 4 practice trials with one step in each direction, a random sequence of 20 trials in which the directions of word and shape never match commences. Participants step with the left foot to the left panel, with the right foot to the right panel and with either foot to the front and back panels. If participants step on a wrong panel they have to repeat the trial until they step correctly. In each of the three tests, reaction time measured from stimulus occurrence to movement initiation (lift off); movement time measured from movement initiation to step finalisation (step down) and stepping errors are measured.

*Functional mobility* will be assessed with the SPPB which includes tests of side-by-side, semi-tandem, and tandem standing, walking speed over 3 metres, and time to complete five chair rises.

*Quality of life* will be measured with the SF-12 _V_2 [44] and ICECAP-O [45] instruments at 3 and 12 months

### Mid-term and follow-up assessments

Physical activity and other leisure time activities will be measured at 6 months via telephone interviews. This is to monitor changes among participants in activities that can enhance performance on tests, apart from dancing. The self-completed quality of life questionnaire will be mailed to participants after 3 months from randomisation. All baseline measurements will be repeated at 12-month follow-up. Researchers performing all measurements will be blinded to group allocation and participants will be asked not to reveal to researchers which group their village was in.

#### Sample size

It is in the control group will be 0.85 per person per year, as in a previous fall prevention study in retirement villages [46], and that falls per person follow a negative binomial distribution with dispersion parameter 0.79 (Lord, personal communication). To have 80% power to detect a 37% reduction in falls in the dance group, as reported for group-based tai chi [8], with a two-tailed 5% significance level, an individually randomised trial would require 171 per group [47]. With a hypothesized intracluster correlation coefficient of 0.015 [48] and a mean of 22 participants recruited per village (cluster size), the required sample size per group is 225 [49]. Assuming 90% completion of falls diaries (10% loss to follow-up) we need 250 participants per group, or 500 in total. To recruit at least 500 participants with a cluster size of 22, we will need to recruit 24 villages.

This sample size will be sufficient to detect a moderate *Cohen*’*s d* effect size of 0.35 for the TMT part B, as was found for older people engaged in aerobic exercise versus yoga [29]. To achieve 80% power to detect an effect size of 0.35, we would require 217 participants per group, assuming the same clustering design effect as above and that 70% of participants complete the follow-up cognitive tests.

#### Statistical analysis

Intention-to-treat analysis will be conducted. We will use negative binomial regression with a random effect with robust standard errors (to allow for clustering) [50] to estimate the magnitude of the intervention effect on number of falls. Generalised linear models with a random effect with robust standard errors will be used to estimate the intervention effect on cognitive measures [51]. The product-of-coefficient test and bootstrap methods will be used to estimate the mediating effects of physiological and cognitive measures of the intervention effect on falls [52]. All models will be fitted both unadjusted (primary analysis) and adjusted for PPA and TMT-B (the minimisation variables), age and sex, education, country of birth, main occupation, use of medications, chronic conditions, falls history and for baseline values for secondary outcomes other than cost. Both unadjusted and adjusted analyses will be reported. All statistical analysis will be blind to group identification. Sensitivity analysis will be used to assess the differential effects (if any) of different strategies of handling missing data (i.e. last observation carried forward, single imputation, multiple imputation) [53].

### Economic evaluation

Cost-effectiveness and cost-utility analyses based on patient and cluster level trial data will be performed. Additional within-trial economic outcomes include total costs of the social dancing program, total costs of the comparator group, $ per fall-rate (i.e. the fall rate in the control group minus the fall rate in the dancing group, to account for person-time at risk); and the $ per mean number of falls prevented (mean number of falls in the control group minus the mean number of falls in the dancing group) [54]. Costs for total health care resource utilisation, rather than only falls-related healthcare utilisation will be collected monthly for 12 months. Participants will report any inpatient hospital admissions, emergency department visits, ambulance trips, doctor and allied health visits, prescribed medications, home care / nursing care, nursing home or long term care admission in their falls diaries. The cost for each of these items will be taken from the relevant source (e.g., Australian Medicare Benefits Schedule fee for service, and Pharmaceutical Benefits Scheme for medications). Participant out-of-pocket costs and care provided by family and /or friends will not be included. All costs will be measured in 2013 Australian dollars, and discounting will not be applied. A cost-effectiveness acceptability curve (CEAC) will be presented showing the probability of cost-effectiveness of social dancing at different willingness to pay levels [55].

*Cost per fall prevented* will be measured using an incremental cost effectiveness ratio (ICER) of the difference in dance program costs divided by the difference in dance program benefits (i.e. falls prevented) between the intervention and control groups. Cost will be calculated from a health system perspective and will include the cost of the dance program, cost of falls, and cost of medical treatments. Cost and benefits will be measured at 12 months with the formula:

$$\begin{array}{rl} \mathrm{ICER}= & \mathrm{Costofnewintervention}\left(\mathrm{danceprogram}\right)\\ & \frac{-\mathrm{Costofcomparator}\left(\mathrm{nodancing}\right)}{\mathrm{Benefitofnewintervention}\left(\mathrm{dance}\right)-}\\ & \mathrm{Benefitofcomparator}\left(\mathrm{nodance}\right) \end{array}$$

*Cost per QALY gained* will be calculated as above based on utility estimates from the SF-12 quality of life instrument [56]. Costs and benefits will be measured at 12 months from a health system perspective.

#### Handling uncertaint

Bootstrapping will be used to estimate a distribution around costs and benefits, and to estimate the confidence intervals around the incremental cost-effectiveness ratio [57]. Cost data are often right skewed due to a few high-cost events such as a hip fracture, transfer to a high-care level nursing home, or hospital admission. A log transformation of cost data, a gamma distribution, or bootstrapping will be applied if the data are not normally distributed. One-way sensitivity analysis will be conducted around key variables and a probabilistic sensitivity analysis will be conducted to estimate joint uncertainty in all parameters. Total hospital costs will be presented in the base case analysis and fall-related hospital costs will be explored in sensitivity analyses.

## Discussion

This trial will be the first to test the effectiveness of typical community social dance programs on falls and age-related cognitive decline. Exercise regimens have previously been shown to protect against cognitive decline in older adults [58,59] and, in the only prospective study that examined the protective effect of specific activity type, dance was shown to reduce the risk of dementia by 35% [60]. Although the type of activity and dose to prevent cognitive decline is far from being defined, balance training of at least 120 minutes per week is considered as best practice for fall prevention [10], which is the ‘dose’ tested in this study. Further, the balance training should challenge balance abilities [10]. While the improvement in balance measures through dance may be equivalent to or only marginally better than that in specific balance training exercise, dance has the potential to address a much wider range of fall risk factors than balance training alone due to its synergetic sensorimotor, rhythmical and cognitive demands.

This study will broaden the knowledge for best practice physical activity for fall prevention, as opposed to exercise (i.e., structured repetitive activity to improve dimension of fitness). The Public Health Physical Activity recommendations for older adults issued by the American College of Sports Medicine and the American Heart Association in 2007 highlight the fact that there is limited research on which to base fall prevention recommendations about type and amount of physical activity, hence recommendations are limited to exercise [61]. Currently, with the exception of Tai chi, it is unknown whether other prevalent physical activities provide sufficient challenge to balance abilities. Walking, golf, bowls, cycling, dance or yoga, all are common leisure activities among older people [62] but their effectiveness in relation to neuromuscular age-related decline is yet to be shown.

The relative effectiveness of different dance styles has been suggested as a future area of research [13]. In this study we assume that both dance styles (traditional and ballroom) are equally effective because they share similar principles: movements are synchronised to music and organised into spatial patterns which tend to be modular in organisation (i.e., composed of discrete sections that are repetitive). For example, the waltz rhythm appears in several folk and ballroom dances. Although there will be approximately 100 participants in each of the dance styles, this study is not powered to ascertain differences between the effects of the two dance styles on fall rates. However, it may be possible to explore differences between their effects on continuous outcome measures.

Many interventions are effective in a pseudo-laboratory setting but lose purchase in the “real world”. Dance compares favourably with other exercise programs in terms of dropout rates. In a comparison of line dancing with Tai chi in eight senior centres in Canada, the average dropout rate was significantly lower (10%) in dance classes than Tai chi classes (23%) [63]. Also, about 40% of those who joined line dancing were motivated by ‘fun’ and 29% by ‘social reasons’, compared to 9% and 1% respectively for Tai chi. This 10% drop-out rate in dance classes is also considerably lower than the average drop-out rate from other senior-specific programs (e.g. cardio, strength, osteo, diabetes), which are reported to be on average 26% after 3 months and 49% after a year. It has also been reported that seniors who switch between activity programs (~21%) over a 3-year period are more likely to maintain exercise participation, suggesting variety helps sustain physical activity behaviour [64]. Variety is intrinsic to dance, which does not suffer from the boredom associated with repetitive exercise training, not only due to the variety of steps, styles and music, but also because it is a social activity. For example, in NSW, a folk dancing group in a culturally diverse suburb of Sydney was established in 1989 and after ten years the group was still running autonomously and survived the departure of its founder [65], suggesting long-term sustained behaviour.

Current estimates indicate that the population prevalence of dance participation among older Australians is low; in all Australia Exercise Recreation and Sport Surveys only 1.6% of older men and 2.8% of older women reported participation in social dancing in the past 12 months. [62] In the New South Wales Fall Prevention Survey slightly higher percentages (2.5% and 4% for men and women respectively) reported they danced in the past week [66]. In other countries the reported level is higher, such as in Canada where 10% of older adults participate regularly in social dancing [67]. The low prevalence in Australia can be partially explained by poor accessibility to dance venues and a lack of classes that cater for older adults. It has been noted that participation in physical activity programs, including dance, is higher within Australian retirement villages than in the community [68]. A primary reason for conducting the study in retirement villages was ease of access to a venue. At the completion of this study, we will be able to assess the potential for widespread dissemination. If dance proves to be efficacious, the challenge ahead would be to find ‘in-built’ mechanisms for dissemination such as the enhancement of dance organisations, a GP referral system [69], and strategies for minimising out-of-pocket expenses and maximising accessibility to venues.

## Conclusion

This study aims to determine the effectiveness of social dance as a fall prevention strategy and its potential in the prevention of cognitive declines. It offers a novel approach to balance training for older people, by examining an enjoyable activity that is holistic in execution. As a community-based approach to fall prevention, dance offers older people an opportunity for greater social engagement, thereby making a major contribution to healthy ageing. Providing diversity in exercise programs targeting seniors recognises the heterogeneity of multicultural populations and may further increase the number of taking part in dance classes.

## Competing interests

The PPA (NeuRA FallScreen) is commercially available through Neuroscience Research Australia.

## Authors’ contributions

DM conceptualised the study, led the grant application, registered the trial and drafted the first version of the manuscript. EM manages the execution of the study according to the study protocol, contributed to trial registration and edited the first draft of the manuscript. BC, KJA, EC, CR, JMS, CS, SRL each contributed to trial methodology in their area of expertise, provided comments and editing of the second draft. RLM conceptualised the economic evaluation of the study, drafted the economic evaluation section of the manuscript including selection of the references and commented on the second draft. All authors approved the last version of the manuscript.

## Pre-publication history

The pre-publication history for this paper can be accessed here:

http://www.biomedcentral.com/1471-2458/13/477/prepub
